# The Attitudes of Healthcare Professionals in an Autonomous Community in Spain towards Paediatric Influenza Vaccination

**DOI:** 10.3390/vaccines12060599

**Published:** 2024-05-31

**Authors:** Jaime J. Pérez-Martín, Antonio Iofrío de Arce, Matilde Zornoza-Moreno

**Affiliations:** 1 Prevention and Health Protection Service, Regional Ministry of Health, Ronda de Levante, 11, 30008 Murcia, Spain; jaimej.perez@carm.es; 2 Murcia-El Ranero Primary Care Centre, Murcia Health Service, Paseo Duques de Lugo, 30009 Murcia, Spain; antonio.iofrio2@carm.es

**Keywords:** influenza vaccines, healthcare professional, attitude of healthcare professionals, intramuscular vaccine, inhaled vaccine

## Abstract

In the 2022–2023 influenza season, three autonomous communities anticipated the document approved by the Public Health Commission recommending influenza vaccination for all children aged 6 to 59 months. The primary objective of this study was to evaluate the attitude of healthcare professionals towards the first universal vaccination campaign in our region, as well as the acceptability of the vaccines used and their attitude towards pilot school vaccination. This was a cross-sectional, survey-based, descriptive study. All healthcare professionals involved in the campaign were invited to participate. Overall, 91.9% of surveyed professionals thought that influenza vaccination from 6 to 59 months was important or very important, and 89.8% had previous experience regarding the intramuscular vaccine. Healthcare professionals rated the intranasal vaccine significantly more positively, but there were no differences when asking about each vaccine without comparison. The inhaled vaccine was preferred by 97.5% for the following campaign. Pilot school vaccination had a 75% acceptance rate. The inhaled vaccine was preferred by most professionals, and pilot school vaccination was highly accepted and independently associated with the importance of vaccination as considered by physicians, being a medical doctor, and participation in the pilot programme.

## 1. Introduction

Influenza is a constant threat that causes major morbidity and mortality in the world and is responsible for one million serious cases annually in children under 5 years of age worldwide. It represents a huge public health problem with high socio-economic implications [1].

In 2012, the World Health Organisation (WHO) [2] included children aged 6 to 59 months as a target population for influenza vaccination because of the high burden of disease in this age group. In the same year, the European Centre for Disease Prevention and Control (ECDC) [3] produced a technical report in which it took a favourable position on vaccination in this age group. The Advisory Committee in Immunization Practices (ACIP) in the USA included universal influenza vaccination for children aged 6 to 23 months in the year 2004 [4] and for those aged 24 to 59 months later in 2006 [5]. More than 70 countries now include influenza vaccination in their childhood and adolescent vaccination schedules [6]. 

In Spain, the greatest burden of disease occurs in the group of children under 5 years of age, both due to health resource use and associated morbidity. Regarding incidence, the most affected age groups were those under 15 years of age, considering both the last season prior to the COVID-19 pandemic (2019–2020) and the rate in the previous ones. The maximum weekly incidence rate was reported in the 0–4 years group, even exceeding the incidence of the 5–14 years group. It should be noted that in the 2013–2014 to 2019–2020 seasons, the 0-to-4 years age group suffered an average of 4239 hospital admissions, of which 822 were considered serious hospitalisations, and 249 Intensive Care Unit (ICU) admissions and eight deaths were recorded [7]. Because of this, in October 2022, the Public Health Commission approved the document “Influenza vaccination recommendations for children aged 6 to 59 months” [8], previously approved by the National Immunisation Technical Advisory Group in July 2022, thus culminating the work that began years before, in 2019, which was interrupted by the COVID-19 pandemic. It recommends the universal vaccination of all children aged 6 to 59 months, to protect them against their high burden of disease, starting in the 2023–2024 season. This recommendation was included in the common lifelong vaccination schedule for 2023 [9]. 

Within the framework of the Spanish state, there is a particular territorial organisation by regions, called autonomous communities (ACs), which have been endowed with certain autonomy and self-governance. Each AC has its own unique characteristics and competencies, especially within the healthcare system; thus, patient management is usually different among ACs, including vaccination schedules and recommendations. Three Spanish ACs, Andalusia, Galicia, and the Region of Murcia, already started universal vaccination in this age group in the 2022–2023 season. The Region of Murcia, which represents approximately 4% of the Spanish population [10] was the only Spanish AC to use the live attenuated intranasal vaccine (LAIV) for children aged 24 to 59 months, without any contraindications for this vaccine. In this campaign, the inactivated intramuscular vaccine (IIV) was used for children aged 6 to 23 months or for those with contraindications to the use of LAIV [11]. In its immunisation schedule for the year 2023, the Vaccination Advisory Committee of the Spanish Association of Paediatrics preferentially recommends the intranasal influenza vaccine from the age of 24 months onwards [12]. 

In this first campaign, the coverage target set in the three ACs was 50%, a relatively ambitious target for a seasonal vaccination campaign restricted to certain months of the year. In this regard, the communication, information, and recruitment strategies carried out by Public Health Authorities, as well as the attitude of healthcare professionals towards influenza vaccination due to their informative and active recruitment work, are fundamental to achieve the vaccination coverage target set. Therefore, the primary objective of this study was to evaluate the attitude of healthcare professionals towards the first universal influenza vaccination campaign in children aged 6 to 59 months in the study AC, the Region of Murcia, as well as the degree of acceptability of each of the influenza vaccines used. The secondary objective was to evaluate the attitude of healthcare professionals towards the pilot school influenza vaccination campaign and the possibility of its widespread implementation.

To our understanding, this study provides highly valuable information about healthcare professionals’ attitude towards universal paediatric vaccination because of the novelty of the programme in our country. A detailed analysis of the opinion provided by the different types of healthcare professionals involved in vaccination campaigns may be of high interest for other Spanish or even European authorities, encouraging them to promote similar programmes.

## 2. Materials and Methods

### 2.1. Study Framework

The FLUTETRA study is a cross-sectional descriptive study to assess the attitude of healthcare professionals providing paediatric care (doctors and nurses) towards childhood influenza vaccination in the Region of Murcia. Once the vaccination campaign ended, an online questionnaire was sent by e-mail three times, seven days apart, as a reminder to all primary care healthcare professionals, both in the public and private settings. 

Aiming at the highest possible participation in the study, only two inclusion criteria were established: (i) to be a healthcare professional providing paediatric care, including paediatricians, family doctors, and nurses, within the primary care setting of the AC under study, the Region of Murcia, and (ii) ticking the box to give consent to participation in the study. No exclusion criteria were defined.

The analysis was only performed among those healthcare professionals who had directly or indirectly participated in the vaccination campaign per se, providing counselling or recruiting patients. 

### 2.2. Variables

After agreeing to participate in the study, healthcare professionals completed the questionnaire designed ad hoc (provided as a Supplementary File), which gathered the following information about the responders: sociodemographic variables, type of healthcare professional, attitude towards influenza vaccination, assessment of available products, and factors associated with school influenza vaccination.

A 5-point Likert scale was used to score the survey from 1 (lowest score) to 5 (highest score). The item evaluated and the response categories vary depending on the question asked (see Supplementary File).

### 2.3. Statistical Analysis

Statistical analysis was performed with SAS v9.4 software through the SAS Enterprise Guide v8.3 interface. 

For the descriptive analysis of qualitative variables, frequency distribution tables and percentages were used. Data analysis was performed both overall and stratified by profession, establishing a nursing group and a medical group. 

To assess the comparison between qualitative variables, the Chi-square test was used when applicable, and Fisher’s exact test was used for those cases of Chi-square non-applicability (i.e., for low frequencies). 

Additionally, a post hoc multivariate logistic regression analysis was performed to determine if the healthcare professional’s characteristics were independently associated with their opinion about the utility/feasibility of extending school vaccination through the whole Region of Murcia in the next season vaccination campaign. For this purpose, the following covariates were assessed: the degree of importance of paediatric influenza vaccination, involvement in the paediatric vaccination of children from 6 to 59 months, age, profession, sex, prior experience in influenza vaccination, participation in the pilot experience, LAIV ease/convenience of administration, and LAIV general rating. Variables with *p* < 0.2 in the bivariate logistic regression analysis were considered significant and included in a multivariate model with a stepwise selection method, and the odds ratio (OR) and 95% CIs were calculated. Test results with *p* < 0.05 were considered statistically significant. 

### 2.4. Ethics

This study was approved by the Ethics Committee for Investigation with Medicinal Products of Hospital Clínico Universitario Virgen de la Arrixaca-Area 1 of the AC where the study was conducted. Participants marked a first mandatory box to indicate the acceptance of their participation in the study after due information was given prior to completing the online questionnaire.

This study was conducted in accordance with Good Clinical Practice guidelines and the regulations contained in the Declaration of Helsinki, which are included in the current legislation on biomedical research.

## 3. Results

The study survey was provided to a total of 1140 professionals, of which 339 answered the online survey, meaning that the response rate of the survey was 29.73%. Of the total responders, 284 professionals (88.47% of those who answered, 95% CI 85.07–91.87%) declared that they participated in the vaccination campaign, both regularly and occasionally. Only the records of involved professionals (n = 284) were considered for this analysis, which means 28.16% of the total of 1140 professionals. The responder’s profile was a female professional, aged 40–49 years, with regular experience in influenza vaccination and with a higher proportion of nurses. The detailed sociodemographic description of the participating professionals is summarised in Table 1, with statistically significant differences (*p* < 0.001) between doctors and nurses with respect to age. No statistically significant differences were found regarding other sociodemographic variables, such as sex and previous experience with influenza vaccination.

Among the surveyed population involved in vaccination, most of the nursing group was paediatric nurses (54.4%), followed by family nurses (28.7%), school nurses (16.4%), and family and paediatric nurses (0.5%). Regarding the medical group, only two types of specialists were involved: paediatricians (80.5%) and family and community doctors (19.5%).

When asked about the importance of influenza vaccination in children aged 6 to 59 months in terms of disease burden, 91.9% of the professionals assigned a score of 4 or 5 on the Likert scale, with statistically significant differences (*p* < 0.001) with regard to profession, with a higher proportion among doctors (n = 80; 97.6%) compared to nurses (n = 181; 89.6%).

**Figure 1:**
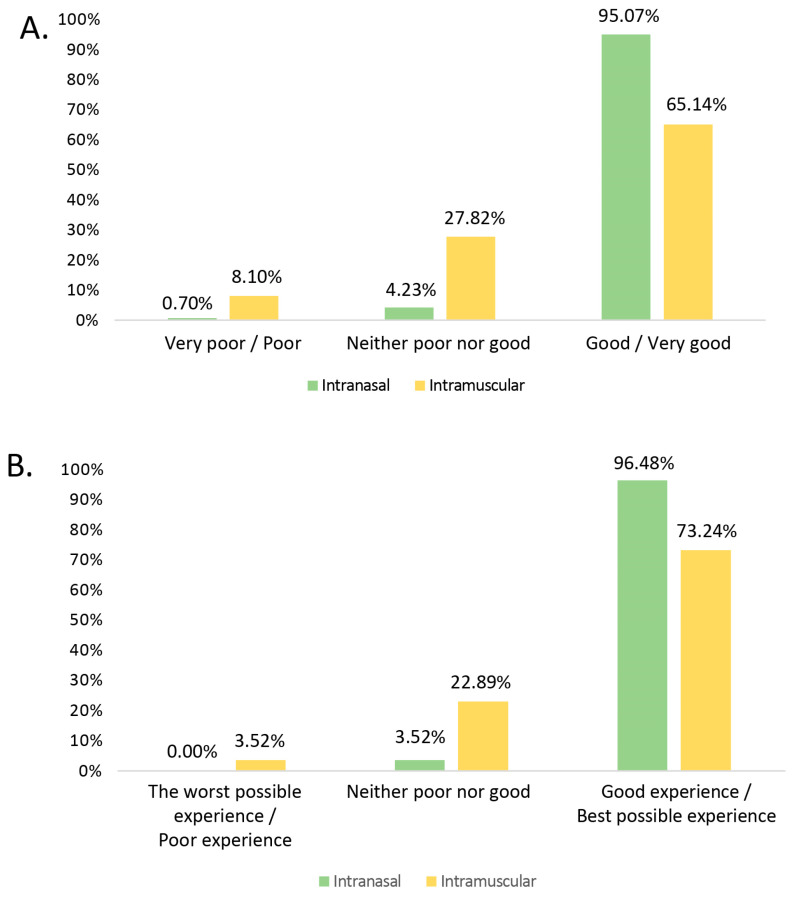
The percentage of healthcare professionals who rated the intranasal vs. intramuscular vaccines used in the vaccination campaign in a comparative analysis according to a Likert scale, where 1 is the lowest possible score and 5 is the highest. (**A**) represents the scores given to “Ease/convenience of administration” (survey question 10, Supplementary File). For this analysis, scores of 1 or 2 on the Likert scale were translated as the very poor/poor, scores of 3 as neither poor nor good, and scores of 4 or 5 as good/very good rating (*p* < 0.001), and (**B**) represents the scores given to “General rating of professional’s experience with the vaccine” (survey question 11, Supplementary File). For this analysis, scores of 1 or 2 on the Likert scale were translated as the worst possible experience/poor experience, scores of 3 as neither poor nor good experience, and scores of 4 or 5 as good experience/best possible experience (*p* < 0.001).

Regarding the assessment of the ease/convenience of administration of the type of vaccine administered, the mean (±SD) scores on the Likert scale obtained for intranasal and intramuscular vaccines were 4.65 ± 0.6 and 3.8 ± 0.97, respectively. A high percentage of respondents appreciated the ease/convenience of administration of the intranasal influenza vaccine, with significantly higher scores (*p* < 0.001) in the overall rating as compared to intramuscular vaccines (Figure 1).

**Figure 2:**
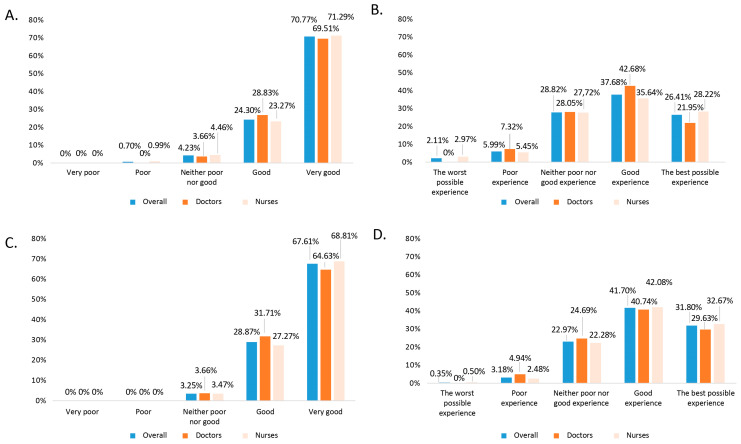
Overall 5-point Likert scale score (blue) and comparing by professional category (doctors in dark orange vs. nurses in light orange). (**A**) Ease/convenience of intranasal vaccine administration (*p* = 0.013 in comparison by professional group). (**B**) General rating of professional’s experience with vaccine (*p* > 0.05 in comparison by professional group). (**C**) Ease/convenience of administration of intramuscular vaccines (*p* < 0.001 in comparison by professional group). (**D**) General rating of professional’s experience with vaccine (*p* = 0.001 in comparison by professional group).

In addition, both ease/convenience and the overall assessment of each of the vaccines in the study population were evaluated in a comparative analysis by professional group, as well as the percentages of scores according to the Likert scale (Figure 2). Overall, the mean scores were 4.64 ± 0.55 for intranasal vaccine and 4.01 ± 0.84 for intramuscular. No statistically significant differences were found between professional groups.

With regard to the preference for using the intranasal vaccine in children aged 24 to 59 months for the next campaign in those cases indicated by the vaccine’s summary of product characteristics [13], 97.5% of the professionals affirmed that they preferred it (100% of doctors and 96.5% of nurses), with no statistically significant differences by professional category.

**Figure 3:**
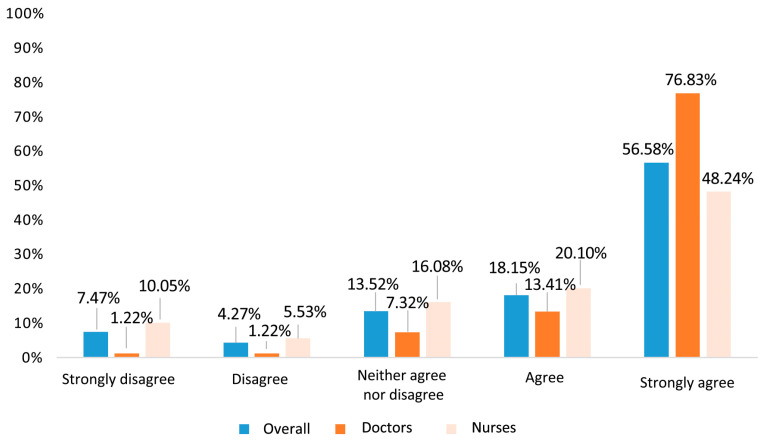
Percentage of healthcare professionals assigning Likert scale scores to question of usefulness/feasibility of extending school influenza vaccination to all schools overall among healthcare professionals and comparatively by professional category (*p* < 0.001 in comparison by professional group).

Only 13.8% (95% CI 10.1–17.5%) of the surveyed professionals had participated in the pilot school vaccination experience. All professionals, both participants and non-participants in the pilot, were asked about the usefulness/feasibility of extending school influenza vaccination to children under 3 and 4 years of age, and most of them gave scores of 4 or 5 (74.7%). Statistically significant differences by professional category were observed (*p* < 0.001), as shown in Figure 3. Stratified according to participation, a significantly higher percentage (*p* = 0.033) of professionals assigned a score of 4 or 5 (86.4%) in the former compared to the latter category (72.3%).

In addition, participants were asked to give their opinion on the importance of the presence of a medical doctor at school vaccination. The most chosen answer by all participants, independently of professional group, was that it was advisable but not essential (nursing group: 47.2% and medical group: 40.2%). Answers stratified according to professional groups showed that 38.2% of the nursing group judged the presence of doctors as completely essential, and only 13.6% considered that the nursing team can autonomously carry out vaccination. In contrast, medical doctors were of the opinion that the nursing team can autonomously carry out vaccination (36.6%), and their presence is only completely essential for 17.1% of the respondents. Answers stratified by professional group were statistically significant (*p* < 0.001). When the stratification was made by those who had participated in the pilot versus those who had not, regarding the recommendation or essential presence of a doctor (72.7% vs. 77.6%, respectively), despite the lack of statistically significant differences between both groups, the percentage was lower in those who had participated in the pilot. However, within each group, statistically significant differences (*p* < 0.001) were evident by professional category (80.6% in nursing vs. 53.8% in doctors in participants in the pilot, compared to 83.9% in nursing and 62.3% in those who did not participate) and also lower in those who took part in the pilot experience.

When asking about the type of vaccine used in school vaccination (intranasal or intramuscular), the vast majority considered the intranasal vaccine as the vaccine of choice for school vaccination (90.6%), evaluating it as recommended or essential for it. Only 5.0% considered the choice of the vaccine to be administered as indifferent, and the rest considered the use of the intramuscular vaccine as advisable or essential.

**Figure 4:**
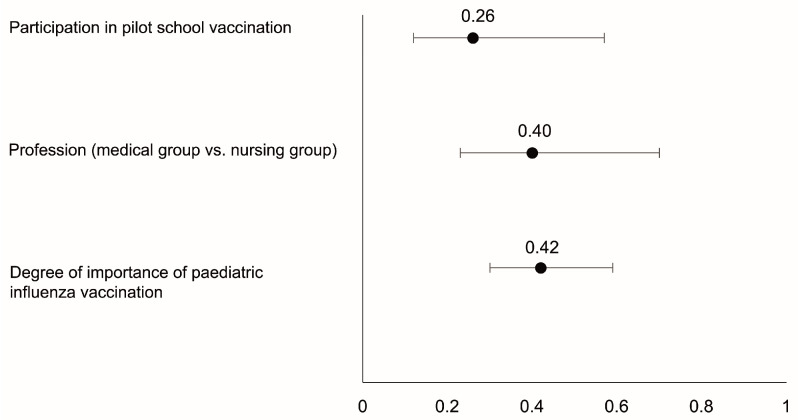
Factors independently associated with a favourable opinion of extending the school vaccination programme for the next season. The *x*-axis represents the odds ratio of each covariate analysed in the multivariate analysis (*y*-axis) along with the 95% CI.

According to the logistic regression, three covariates were independently associated with a favourable opinion of extending the pilot school vaccination programme. A one-point increase in the degree of importance of paediatric vaccination (as considered by the healthcare professional) increased the favourable opinion by 0.42 (odds ratio, OR), as well as belonging to the medical group (OR = 0.40). Similarly, participation in the pilot vaccination school programme was considered independently associated with the favourable opinion for extending the school programme (OR = 0.26). The univariate and multivariate models extracted from the logistic regression analysis and the odds ratio are shown in Table 2 and Figure 4, respectively).

## 4. Discussion

Healthcare professionals are one of the most important sources of information for families in making decisions about vaccination [14,15,16]. This is even more important when discussing a seasonal vaccination campaign and a new vaccination strategy with a vaccine, such as the attenuated intranasal vaccine discussed here. This vaccine set an unprecedented experience in the Region of Murcia and Spain during the 2022–2023 vaccination campaign due to its characteristics and route of administration. 

Our study elicited responses from 82 physicians (paediatricians and family doctors) and 202 nurses (Table 1). This study found that 91.9% of surveyed professionals thought that influenza vaccination in children aged 6 to 59 months was important or very important, a view shared by doctors and nurses, with a statistically significant difference between them, which is probably not of practical relevance (97.6% vs. 89.6%). Similar results have been described in Quebec, where more than 90% of professionals agreed with influenza vaccination [17], or in the USA, where 84% of paediatricians shared the opinion of the importance of vaccination [18]. These data contrast with the results obtained in a study carried out in Australia, which found a lack of awareness of the seriousness of influenza in children [19]. The positive attitude detected among our professionals may be because of the agreement between health authorities [8] and scientific societies on the recommendations [20] and probably also because of the accumulated experience of childhood influenza vaccination in other countries for years. 

From our point of view, it was necessary to discuss similarities or discrepancies in the attitudes of HCPs toward influenza vaccination in children and towards SARS-CoV-2 vaccination, considering the importance of the latter in recent years for both medical professionals and society as a whole. No specific studies evaluating the acceptance/hesitancy of HCPs of paediatric vaccination against COVID-19 in Spain were found, but overall, the vaccine hesitancy among HCPs was much higher, specially before the vaccine’s availability [21,22]. These differences between attitudes regarding influenza or COVID-19 may be easily attributed to the complicated pandemic context, which included mistrust in government and institutions and the rapid development of the vaccine using novel technologies [23]. However, we should also keep in mind that attitudes towards both vaccines should not necessarily be similar either, as the burden of disease in children is very different in influenza and COVID-19.

Although universal paediatric influenza vaccination started in the 2022–2023 season, 89.8% of the professionals had experience of influenza vaccination with the intramuscular vaccine, while the inhaled vaccine had been scarcely available previously. When asked about ease/convenience of administration, 95% said that the intranasal vaccine is easy or very easy to administer, compared to 65.1% for the intramuscular vaccine (Figure 1). Similarly, when rating their overall experience with the vaccines, 96.5% rated the inhaled vaccine positively or very positively, compared to only 73.2% for the intramuscular vaccine. Regarding the inhaled vaccine, it is also remarkable that only 0.7% rated the convenience of its administration negatively, which would support the simplicity of its use, given that this is the first season of widespread administration, and it is expected that the ease of use will increase as experience with this vaccine increases throughout the coming seasons. This difference is illustrated when asking comparatively about the two vaccines, but it is not observed when asking about each of the vaccines without comparing them (Figure 2), as both vaccines score positively or very positively, especially in terms of ease of administration. The intranasal vaccine was preferred by 97.5% of the professionals, who stated that they would prefer it for the next vaccination campaign.

Studies comparing the two vaccines are rare, and ours is one of the few to address this issue. In a study conducted in Quebec [17], more than 90% of the professionals considered that the intranasal attenuated vaccine had been well received, both by parents and by themselves, and 57% rated the ease of administration very positively, a figure that was higher in our data (71%). 

Another possible consequence of the preference for one vaccine over another is an eventual increase in vaccination rates in places where the vaccine is available. However, estimating the influence of the type of vaccine administered on the vaccination rate is complex, since this is influenced by multiple factors. In fact, the data available following the withdrawal of the intranasal vaccine in the US in the 2016–2017 season, which made it possible to estimate the change in vaccination rates compared to the previous season when the attenuated vaccine was available, are discordant. One study found no change in vaccination rates [24], while another estimated a decrease of 1.6%, with a lower tendency to vaccinate in the second season among the most socially disadvantaged [25].

Our study additionally addresses the acceptability of school vaccination among professionals in the Region of Murcia following a pilot study carried out in 24 schools. In this regard, school influenza vaccination programmes have demonstrated an increase in vaccination rates as well as being a valid instrument for mass vaccination in countries such as the USA [26,27], overcoming one of the possible barriers, namely additional visits to the healthcare centre that require both time off work for legal guardians and time off school for children [28]. Furthermore, it should also be considered that childhood influenza vaccination in Spain will mean almost one million additional visits to healthcare centres for children aged 6 to 59 months (estimating a vaccination rate of 60%), limited to a short period of time of approximately 3 months (estimated vaccination campaign duration) based on the National Statistics Institute [INE] data [10]), so incorporating alternative vaccination points can be very useful to avoid overloading the primary healthcare system. School vaccination programmes have shown that the cost per dose of vaccine administered can be lower [29] and can lead to cost savings when considering the cost of working hours lost by legal guardians for the vaccination of children [30] and can therefore contribute not only to increasing accessibility and vaccination rates but also to the efficiency of the system. Furthermore, according to a study published in Eurosurveillance in 2014 with data from the United Kingdom on vaccination against human papillomavirus in adolescents, the strategy of school vaccination also reduces inequities [31]. Another advantage of school vaccination is that it allows for early and rapid vaccination, allowing for high vaccine coverage in children to be vaccinated in just two weeks. This enables us to be prepared for when the flu epidemic season arrives. This is especially important in an epidemic disease that may present early, such as what occurred in the United Kingdom in the 2019–2020 season [32].

In the Region of Murcia, school influenza vaccination was piloted during the 2022–2023 season in 24 schools. For this purpose, families were sent a letter with information on intranasal influenza vaccination and its contraindications, requesting consent from parents/legal guardians and a prior review of the children’s medical records by the vaccination teams. In the first four centres evaluated, the vaccination rate was increased by 22.5% on a single day of school vaccination [33], so it seems pertinent to obtain the professionals’ opinion before extending the programme. When asked about the extension of the pilot programme with the intranasal vaccine to the entire population of children aged 3 and 4 years, almost 75% agreed or strongly agreed (with greater support from doctors, 90%, but also with significant support from nurses, 68.3%). This support was greater among professionals who had participated in the pilot project, which may express that the initial “fear” is overcome after participation in school vaccination (86.4 vs. 72.7%, *p* = 0.03). These data are supported by the results of the logistic regression performed post hoc, which confirmed the fact that giving a high importance to paediatric vaccination directly correlates with a favourable opinion of extending the pilot school vaccination throughout the Region of Murcia for the next season (OR = 0.42). This analysis also highlighted the support from the medical group to the extension of the pilot programme, with a correlation (OR) of 0.40, as well as the association between the participation in the pilot programme and a favourable opinion towards the extension (OR = 0.26). Similar studies have been conducted in the USA. The study conducted by Keane et al. showed less support from paediatricians than seen in Spain, with 73% support for vaccinating children aged 24 to 59 months, which rises to 96% for children aged 5 years and older [34]. In another study, also carried out in a sample of responders from the American Academy of Pediatrics, paediatricians’ support was 86%, although with differences depending on the children’s age and possible risk conditions [35]. In general, support from paediatricians in our setting is somewhat higher, which may be due to the use of the attenuated vaccine, since the US surveys did not propose using only the attenuated vaccine in the school setting. In this regard, another study carried out in the USA associated the use of the attenuated vaccine in schools with an increase in vaccination rates, an increase in the speed of administration, and children who were calmer during the process [36]. 

For many years, adolescents have been vaccinated against meningococcal disease and human papillomavirus at school in the Region of Murcia and, since 2008, in a protocolised manner [37]. As a result of the protocolised school vaccination against human papillomavirus in girls, some healthcare professionals (predominantly nurses) have traditionally demanded the presence of a paediatrician along with the nursing professionals in charge of school vaccination, and this survey was an excellent opportunity to address this issue. Our data revealed that two out of three surveyed healthcare professionals do not consider the presence of a doctor necessary, they consider it advisable but not essential, or they have a neutral opinion. As expected according to the line of opinion that had been observed in the nursing teams dedicated to school vaccination, our data reveal that the presence of physicians is considered completely essential by a higher number of professionals in the nursing group than in the medical group itself (38.2% vs. 17.1%) and vice versa; doctors are more likely to think that the nursing team can autonomously carry out school vaccination compared to nurses themselves (36.6% vs. 13.6%). This can be interpreted as an attempt by physicians to increase the competencies of nursing professionals, considering them sufficiently qualified to carry out the vaccination autonomously, as well as a lower number of immediate adverse reactions expected as it is an intranasal vaccine. Most likely, the need for the presence of a physician on the part of nursing comes from the previous experience with intramuscular vaccination against human papillomavirus in adolescents, so these data are presented as a baseline, which will have to be studied in successive campaigns in which there is more experience in school intranasal influenza vaccination, to see if the presence of a doctor is considered equally or less necessary. In fact, when analysing the data among those who had or had not participated in the pilot, a tendency (not statistically significant) was observed to give less importance to the presence of a doctor in the vaccination. Another plausible hypothesis is that both groups of professionals have too large a workload for taking on this new responsibility and so attempt to share this emerging task with other healthcare professionals, nursing considering school vaccination a task of the primary care team, of which doctors are also part.

Regarding the type of vaccine preferred for administration in the school environment, the results obtained are as expected since for the vast majority of professionals, the vaccine of choice should be the intranasal vaccine due to its ease of administration and being painless, which makes it possible to administer it to young children in schools without incidents.

Some interesting findings suggest that the acceptance of self-vaccination and the recommendation of paediatric vaccination are not always correlated [38,39,40]. We do not have the HCPs’ history of influenza vaccination nor their willingness to be vaccinated against it, but in different recently published papers, we found that the willingness of HCPs to be vaccinated against influenza varies between 52 and 54.9% [41,42], which contrasts with the support for the vaccination of the paediatric population in our study (91.9%). It might be reasonable to expect that the non-responders were less willing to support the vaccination and therefore to refuse to participate in the study; nevertheless, we consider that other reasons for not participating are more plausible such as high workload, a lack of motivation to complete the survey, or even forgetfulness. 

Our study has a few limitations. First, the response rate was 29.7%, despite having sent the information three times, each 7 days apart, which could mean that the respondents were not particularly motivated. However, there are other published studies carried out in the USA that had similar response rates [16,34], in addition to the fact that most of the literature consulted had similar or smaller sample sizes. Second, we found a significant majority of nurses participating and responding to the survey in contrast to doctors, and we must take this into account as a bias in our results, since the attitudes and opinions might differ among professions. As in this manner, to ensure a truthful and substantiated response to the survey, only HCPs that had participated in the campaign were considered for this analysis (n = 284/339), so this participation already implies a positive attitude towards vaccination. Most likely, those HCPs reluctant to recommend the universal paediatric vaccination have not participated in our survey.

We would like to highlight that this study was conducted during the first season of influenza vaccination in the paediatric population in the AC under study (Region of Murcia), and herein, we present the first-obtained results of this program, but this study has been reproduced for the second influenza vaccination season, aiming to compare both campaigns and other campaigns yet to come. As an improvement, the survey used for the second campaign now includes the vaccination status of the HCPs. In addition, we believe that the simultaneous study of support for the vaccination of patients and healthcare workers themselves is an interesting endpoint for future research, since it may provide a different point of view to evaluate healthcare professionals’ positioning regarding influenza vaccination.

## 5. Conclusions

In conclusion, the results of our study show the significant support of healthcare professionals involved in childhood influenza vaccination for the use of a vaccine such as the attenuated intranasal vaccine, which had not been used before, and have achieved a 97% preference rate for the next vaccination campaign. Our data reveal good acceptance for extending the school vaccination programme, with almost 75% of professionals supporting it and considering the intranasal vaccine as suitable for school vaccination. Additionally, we found an association between the degree of importance given to paediatric vaccination, being a medical doctor, and participation in the pilot programme with a favourable opinion for extending the pilot throughout the Region of Murcia.

## 6. Future Directions

There is still a need for information and the training of professionals to strengthen vaccination programmes, as well as further research on their opinions and attitudes, so that programmes can be tailored to their needs, even more so in seasonal vaccination campaigns such as the influenza campaign in the paediatric population, which require a high recommendation to achieve coverage targets.

## Figures and Tables

**Table 1:** Sociodemographic variables of healthcare professionals involved in the 2022–2023 influenza vaccination campaign in the paediatric population aged 6 to 59 months.

		Doctor—N (%)	Nurse—N (%)	Total—N (%)	*p*-Value
Age	<20 years	0 (0.0%)	1 (0.4%)	1 (0.4%)	<0.001
	20–29 years	0 (0.0%)	8 (3.9%)	8 (2.8%)	
	30–39 years	22 (26.8%)	35 (17.3%)	57 (20.1%)	
	40–49 years	19 (23.2%)	75 (37.1%)	94 (33.1%)	
	50–59 years	27 (32.9%)	56 (27.7%)	83 (29.2%)	
	≥60 years	14 (17.1%)	27 (13.4%)	41 (14.4%)	
Sex	Male	22 (26.8%)	48 (23.8%)	70 (24.6%)	0.587
	Female	60 (73.2%)	154 (76.2%)	214 (75.4%)	
Previous experience in influenza vaccination	No	1 (1.2%)	2 (1.0%)	3 (1.1%)	0.076
	Occasional	8 (9.8%)	18 (8.9%)	26 (9.2%)	
	Regular	73 (89.0%)	182 (90.1%)	255 (89.8%)	
Total number of participants		82 (28.9%)	202 (71.1%)	284 (100%)	

**Table 2:** Univariate and multivariate analyses extracted from the logistic regression model used to estimate the healthcare professional’s characteristics independently associated with the opinion of extending the school vaccination programme.

Covariates	Univariate Analysis		Multivariate Analysis	
	*	*p*-Value	OR (95% CI)	*p*-Value
Degree of importance of paediatric influenza vaccination ^(1)^	0.35	<0.001	0.42 (0.30–0.59)	<0.001
Involved in paediatric vaccination of children from 6 to 59 months ^(2)^	88.4%	0.457	-	-
Age ^(3)^		0.363		
<20	4.0 ± 0.0		-	-
20–29	3.9 ± 1.5		-	-
30–39	4.4 ± 1.0		-	-
40–49	4.2 ± 1.2		-	-
50–59	3.9 ± 1.4		-	-
>60	4.1 ± 1.3		-	-
Profession ^(4)^				
Medical group	27.4%	<0.001	0.40 (0.23–0.70)	0.001
Nursing group	72.6%			
Sex ^(5)^				
Female	89.0%	0.143	-	-
Male	11.0%			
General rating of intranasal vaccine ^(2)^	0.19	0.005 **	-	-
Participation in pilot experience ^(2)^	13.8%	0.003	0.26 (0.12–0.57)	<0.001
Ease/convenience of intranasal vaccine ^(1)^				
1–3	3.2 ± 1.6	0.004	0.48 (0.17–1.35)	0.164
4–5	4.2 ± 1.2			
Ease/convenience of intramuscular vaccine ^(1)^				
1–3	4.0 ± 1.3	0.259	-	-
4–5	4.2 ± 1.2			

* Values in the univariate analysis are presented by statistical dispersion measures (mean ± standard deviation), frequency (%), and correlation coefficient, depending on the type of covariate analysed. ** The variable “general rating of intranasal vaccine” was significant in the univariate analysis but was not included in the multivariate analysis due to it being already related to another covariate included in the model. ^(1)^ For these variables, no reference categories are indicated. The values in the univariate analysis represent the change in the dependent variable considering a one-point increase in the numerical covariate (independent); ^(2)^ Reference category: none, ^(3)^ Reference category: >60 years, ^(4)^ Reference category: nursing group, ^(5)^ Reference category: male.

## Data Availability

The data that support the findings of this study are available on request from the corresponding author. The data are not publicly available due to privacy or ethical restrictions.

## Supplementary Materials

The following supporting information can be downloaded at https://www.mdpi.com/article/10.3390/vaccines12060599/s1, Survey questions.
