# Supplementary material for: The Attitudes of Healthcare Professionals in an Autonomous Community in Spain towards Paediatric Influenza Vaccination
Source: Vaccines (Basel). 2024 May 31;12(6):599. doi: 10.3390/vaccines12060599 (PMC11209165; doi:10.3390/vaccines12060599)
Supplement: Supplementary file 1 [file vaccines-12-00599-s001.zip › vaccines-2996770-supplementary.pdf]

Survey questions

1. Have you been involved as a healthcare professional in the influenza vaccination campaign for children aged 6 to 59 months? Yes/No.
2. Age: Under 20 years/20–29 years/30–39 years/40–49 years/50–59 years/Over 60 years.
3. Sex: Male/Female.
4. Profession: Adult Nursing/Paediatric Nursing/School Nursing/Family Doctor/Paediatrician.
5. Please indicate the degree of importance to vaccinate children aged 6 to 59 months against influenza based on the burden of disease, with 1 being the lowest possible score and 5 being the highest: 1/2/3/4/5.
6. Please indicate your previous experience in influenza vaccination: Occasional/Regular/Comprehensive.
7. Please rate the ease/comfort of administration of Fluenz Tetra® (intranasal influenza vaccine), with 1 being the lowest possible score and 5 being the highest: 1/2/3/4/5.
8. Please give an overall rating of your experience with Fluenz Tetra® (intranasal influenza vaccine), with 1 being the worst possible experience and 5 being the best: 1/2/3/4/5.
9. Would you like next season's influenza vaccination to continue with an intranasal vaccine in those age groups for which the SmPC allows it? Yes/No/I am indifferent.
10. Please rate the ease/comfort of administration of intramuscular inactivated influenza vaccines (Influvac Tetra® and Flucelvax Tetra®), with 1 being the lowest possible score and 5 being the highest: 1/2/3/4/5.
11. Please give an overall rating of your experience with intramuscular inactivated influenza vaccines (Influvac Tetra® and Flucelvax Tetra ®), with 1 being the worst possible experience and 5 being the best: 1/2/3/4/5.
12. Have you participated in the pilot school influenza vaccination programme in this campaign? Yes/No.
13. Whether or not you have participated in the pilot school influenza vaccination, do you think it would be useful/feasible to extend school vaccination to the whole Region in the next vaccination campaign, with 1 being the lowest possible score and 5 being the highest? 1/2/3/4/5.
14. To what extent do you consider the presence of a doctor to be essential for school influenza vaccinations? It is not necessary, as school nurses can take over the school influenza vaccination independently/Neutral/Advisable, but not essential/Absolutely essential.
15. In school influenza vaccination for children under 3 and 4 years of age, what importance do you place on the choice of influenza vaccine? I consider it essential that the vaccine to be administered should be intramuscular / I consider it essential that the vaccine to be administered should be intranasal / I consider the type of vaccine to be administered to be of little importance, so I do not choose one or the other / I consider it advisable that the vaccine to be administered should be intramuscular / I consider it advisable that the vaccine to be administered should be intranasal.
